# Perioperative Morbidity and Complications in Patients With an Established Ileostomy Undergoing Major Abdominal Surgery: A Retrospective Study

**DOI:** 10.3389/fsurg.2021.757269

**Published:** 2021-12-08

**Authors:** Alberto A. Uribe, Tristan E. Weaver, Marco Echeverria-Villalobos, Luis Periel, Haixia Shi, Juan Fiorda-Diaz, Alicia Gonzalez-Zacarias, Mahmoud Abdel-Rasoul, Lin Li

**Affiliations:** ^1^Department of Anesthesiology, The Ohio State University Medical Center, Columbus, OH, United States; ^2^Center for Biostatistics, Department of Biomedical Informatics, The Ohio State University Medical Center, Columbus, OH, United States

**Keywords:** ileostomy, complications, abdominal surgery, established, perioperative

## Abstract

**Background:** Recently formed ileostomies may produce an average of 1,200 ml of watery stool per day, while an established ileostomy output varies between 600–800 ml per day. The reported incidence of renal impartment in patients with ileostomy is 8–20%, which could be caused by dehydration (up to 50%) or high output stoma (up to 40%). There is a lack of evidence if an ileostomy could influence perioperative fluid management and/or surgical outcomes.

**Methods:** Subjects aged ≥18 years old with an established ileostomy scheduled to undergo an elective non-ileostomy-related major abdominal surgery under general anesthesia lasting more than 2 h and requiring hospitalization were included in the study. The primary outcome was to assess the incidence of perioperative complications within 30 days after surgery.

**Results:** A total of 552 potential subjects who underwent non-ileostomy-related abdominal surgery were screened, but only 12 were included in the statistical analysis. In our study cohort, 66.7% of the subjects were men and the median age was 56 years old (interquartile range [IQR] 48-59). The median time from the creation of ileostomy to the qualifying surgery was 17.7 months (IQR: 8.3, 32.6). The most prevalent comorbidities in the study group were psychiatric disorders (58.3%), hypertension (50%), and cardiovascular disease (41.7%). The most predominant surgical approach was open (8 [67%]). The median surgical and anesthesia length was 3.4 h (IQR: 2.5, 5.7) and 4 h (IQR: 3, 6.5), respectively. The median post-anesthesia care unit (PACU) stay was 2 h (IQR:0.9, 3.1), while the median length of hospital stay (LOS) was 5.6 days (IQR: 4.1, 10.6). The overall incidence of postoperative complications was 50% (*n* = 6). Two subjects (16.7%) had a moderate surgical wound infection, and two subjects (16.7%) experienced a mild surgical wound infection. In addition, one subject (7.6%) developed a major postoperative complication with atrial fibrillation in conjunction with moderate hemorrhage.

**Conclusions:** Our findings suggest that the presence of a well-established ileostomy might not represent a relevant risk factor for significant perioperative complications related to fluid management or hospital readmission. However, the presence of peristomal skin complications could trigger a higher incidence of surgical wound infections.

## Introduction

Temporary or permanent diverting loop ileostomies are commonly created to protect a distal anastomosis with a high risk of anastomotic leakage, resulting in reduced morbidity and mortality (1). Ileostomies are performed as part of either emergency or elective procedures. Having less viscous effluence at a higher volume is associated with early postoperative complications. Recently formed ileostomies may produce an average of 1,200 ml of watery stool per day (2), while an established ileostomy (longer than 1 year) output varies between 600 and 800 ml per day (3, 4). Excessive fluid loss through the stoma and the inability of the small bowel to preserve sodium, chloride, and bicarbonate may result in life-threatening complications such as acute dehydration, electrolyte imbalance, and acid-base disorder (5–8). Furthermore, the loss of excessive amounts of sodium in stools contributes to chronic sodium depletion and dehydration with secondary hyperaldosteronism as compensatory post-ileostomy adaptation (9). Conversely, patients with long-standing ileostomies often experience hypomagnesemia, decreased absorption of folic acid and vitamin B-12 (9).

Acute or chronic intestinal failure is a long-term complication after ileostomy (7, 8, 10). High-output stoma (HOS) is defined as a stoma with an output of 1–2 L over a 24-h period and may develop within the first-month post-ileostomy up to 16% of patients (2, 11). The most common causes of HOS include, but are not limited to, intestinal resection, partial bowel obstruction, chronic bowel dysfunction (dysmotility disorders, radiation enteritis), intra-abdominal sepsis, steroid withdrawal following surgery for inflammatory bowel disease (IBD), prokinetic drugs, and *Clostridium difficile* infection (2, 12). Dehydration, electrolyte depletion, and acute kidney injury (AKI) are some of the acute complications of HOS, and malnutrition and chronic kidney disease (CKD) are identified as late complications (10, 13–15). The reported incidence of renal impartment in patients with ileostomy is 0.8–20%, and this complication could be caused by dehydration (up to 50%) or HOS (up to 40%) (15). Although, the literature describes dehydration and renal failure as potential complications in this surgical population, there is a lack of evidence if an ileostomy could influence perioperative fluid management and/or surgical outcomes. In addition, there is a lack of information regarding the perioperative consideration and management of patients with an established ileostomy undergoing non-ileostomy related surgeries. Therefore, we designed a retrospective study to assess the incidence of perioperative complications in patients with an established ileostomy undergoing non-ileostomy related surgical procedures.

## Materials and Methods

We conducted a retrospective, single-center, observational chart review of subjects with an established ileostomy who underwent non-ileostomy-related major abdominal surgeries under general anesthesia at The Ohio State University Wexner Medical Center between May 1, 2014, and May 31, 2019. After obtaining the approval of our Institutional Review Board (Office of Responsible Research Practices, The Ohio State University), we accessed the electronic medical records to evaluate eligibility and collect perioperative data from eligible subjects.

### Participants

Subjects aged ≥18 years old with an established ileostomy who were scheduled to undergo an elective non-ileostomy related major surgery under general anesthesia lasting more than 2 h and requiring hospitalization between May 1, 2014, and May 31, 2019, at The Ohio State University Wexner Medical Center were included in the study. In contrast, prisoners, pregnant or breastfeeding women, subjects with CKD before surgery, newly established ileostomies, and surgical procedures lasting <2 h were excluded from the analysis.

### Clinical Outcomes

The primary outcome was to assess the incidence of perioperative complications within 30 days after surgery. The secondary outcomes were to identify the potential risk factors associated with the perioperative complications, including subject demographics and perioperative variables.

### Data Collection and Data Management

The following preoperative information was retrieved from the electronic medical records and collected in a data sheet created as part of our electronic data capture system: demographics (age, gender, weight, height, median body mass index [BMI]), preoperative American Society of Anesthesiologists (ASA) physical status, history of smoking, mental disorders, neurologic disease or any other diagnosed cognitive impairment, alcohol or drug abuse, coronary artery disease, active malignancy, and concomitant medications. The perioperative variables collected included time from the ileostomy creation to the surgery date, type of surgery and anesthesia, length of anesthesia, estimated blood loss (EBL), post-anesthesia care unit (PACU) stay, intensive care unit (ICU) stay (if available), vital signs, significant laboratory results, intraoperative administration of vasoactive drugs and fluid administration, perioperative blood transfusion requirements, diuresis, and ostomy output. In addition, other inpatient variables such as discharge disposition, readmissions, and perioperative complications within the first month after surgery were reviewed.

### Statistical Analysis

Continuous variables of demographic data, clinical characteristics, lab values, and hospital readmissions were summarized using descriptive statistics and expressed as median [interquartile range (IQR)] for continuous variables and frequency (percentage) for categorical variables. All analyses were conducted using SAS version 9.4 (SAS Institute, Cary, NC, USA).

## Results

**Figure 1:**
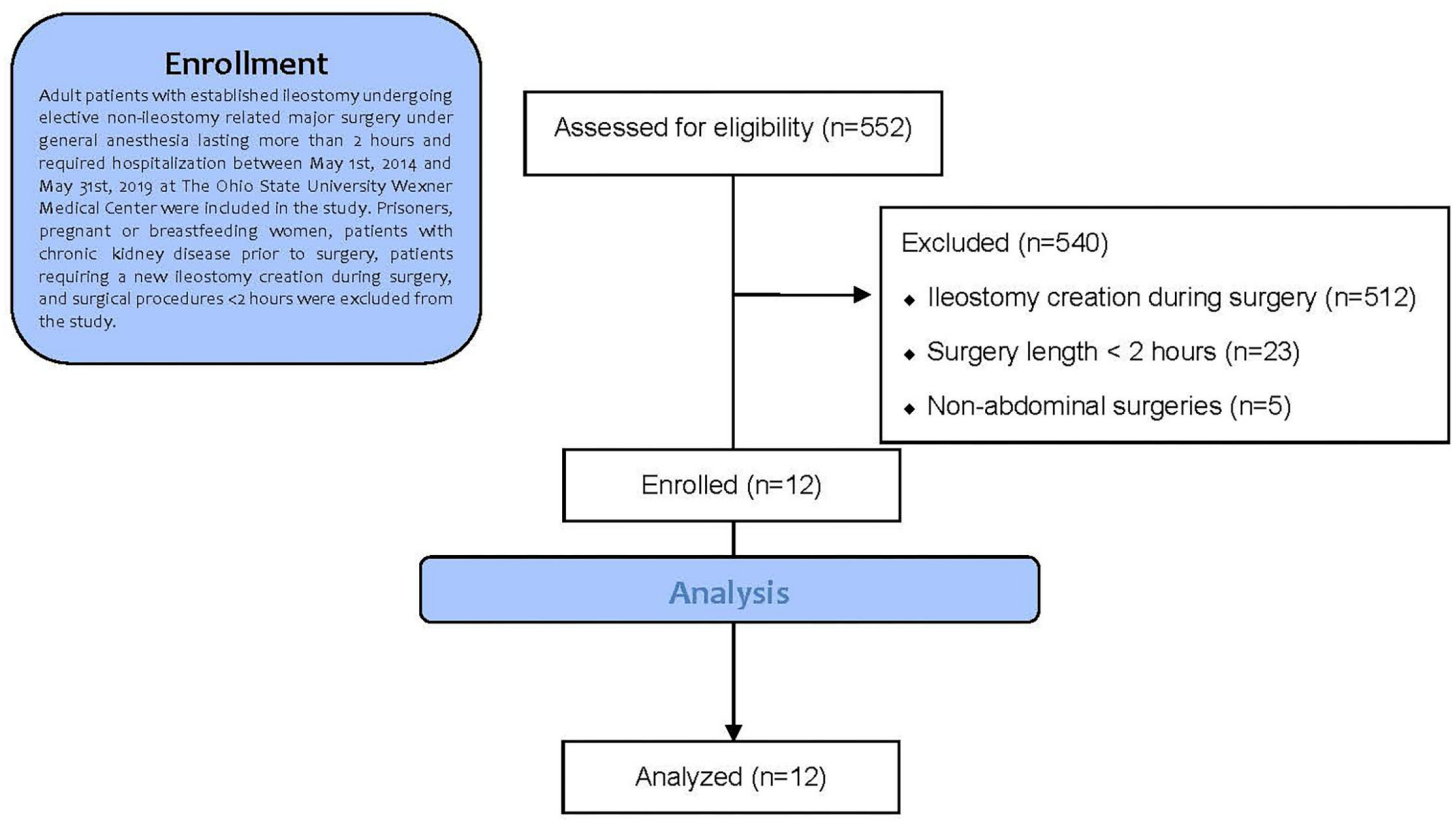
Trial profile according to consolidated standards of reporting trials (CONSORT) guidelines.

We screened 552 potential subjects who underwent non-ileostomy-related abdominal surgery during hospitalization at the Ohio State University Wexner Medical Center between May 1, 2014, and May 31, 2019. Of this subject list, 540 subjects were excluded due to ileostomy creation during surgery (*n* = 512), surgery length <2 h (*n* = 23), and non-abdominal surgeries (*n* = 5). Therefore, a total of 12 subjects were included in the statistical analysis. The Consolidated Standards of Reporting Trials (CONSORT) flow diagram is shown in Figure 1 (16).

In our study cohort, 66.7% of the subjects were men, the median age was 56 years old ([IQR] 48–59), and the median BMI was 26.1 kg/m^2^ (IQR: 21.6, 29.7). All the subjects had an ASA physical status of 3 and the median time from the creation of ileostomy to the qualifying surgery was 17.7 months (IQR: 8.3, 32.6). The most predominant surgical approach was open (8 [67%] subjects). The most common type of open surgery was proctectomy (2 [16.7%] subjects) and the most common type of laparoscopic surgery was ventral hernia repair (3 [25%] subjects).

The most prevalent comorbidities in the study group were psychiatric disorders (58.3%), hypertension (50%), and cardiovascular disease (41.7%). At the time of surgery, 47.7% were current smokers and 16.7% were former smokers; in addition, around 33.3% used another type of drug and 8.3% consumed alcohol. The median surgical and anesthesia length was 3.4 h (IQR: 2.5, 5.7) and 4 h (IQR: 3, 6.5), respectively. The median PACU stay was 2 h (IQR:0.9, 3.1), while the median length of hospital stay (LOS) was 5.6 days (IQR: 4.1, 10.6) (Table 1).

**Table 1:** Demographics and clinical variables.

**Demographics and clinical variables**	***N* (12)**
Age, years, median [IQR]	56 [48, 58.5]
Sex male, *N* (%)	8 (66.7%)
Race, *N* (%)	
White	10 (83.3%)
African American	2 (16.7%)
Hispanic, *N* (%)	0 (0.0%)
Height, meter, median [IQR]	1.8 [1.7, 1.8]
Weight, kilogram, median [IQR]	84 [65.5, 90.8]
BMI, units, median [IQR]	26.1 [21.6, 29.7]
ASA III, *N* (%)	12 (100%)
Social characteristics, *N* (%)	
Current Tobacco use	5 (41.7)
Other drug use	4 (33.3%)
Former Tobacco use	2 (16.7%)
Alcohol use	1 (8.3%)
Comorbidities, *N* (%)	
Psychiatric disease	7 (58.3%)
Hypertension	6 (50.0%)
Cardiovascular disease	5 (41.7%)
Neurological disease	3 (27.3%)
Coronary artery disease	1 (8.3%)
Type of surgery, *N* (%)	
Open Approach	8 (66.7%)
Proctectomy	2 (16.7%)
Whipple	1 (8.3%)
Adrenalectomy	1 (8.3%)
Panniculectomy and reposition of Ileostomy	1 (8.3%)
Hysterectomy	1 (8.3%)
Partial liver resection	1 (8.3%)
Exploratory laparotomy	1 (8.3%)
Laparoscopic approach	4 (33.3%)
Ventral hernia repair	2 (16.7%)
Cholecystectomy	1 (8.3%)
Proctectomy	1 (8.3%)
Length of surgery, hours, median [IQR]	3.4 [2.5, 5.7]
Length of anesthesia, hours, median [IQR]	4.0 [3.0, 6.5]
Length of PACU stay, hours, median [IQR]	2.0 [0.9, 3.1]
Length of Stay, days, median [IQR]	5.6 [4.1, 10.6]
Discharge disposition, *N* (%)	
Home	9 (75.0%)
Nurse facility	3 (25.0%)
Time from ileostomy to surgery date, months, median [IQR]	17.7 [8.3, 32.6]
Postoperative complications, *N* (%)	6 (50%)
Moderate surgical infection	2 (16.7%)
Mild surgical infection	2 (16.7%)
Atrial fib.	1 (8.3%)
Postoperative bleeding	1 (8.3%)
Readmission within 30 days from discharge	2 (16.7%)

*N, number; %, percentage; IQR, interquartile range; BMI, body mass index; ASA, American Society of Anesthesiologists; PACU, post-anesthesia care unit*.

The median and IQRs for intraoperative variables were as follows: fluid administration 3,200 ml (IQR: 2,350, 3,950 ml), fluid balance output 2,275 ml (IQR: 1,815, 2,863 ml), EBL 500 ml (IQR: 50, 650), and diuresis 418 ml (IQR: 300, 581.3). Six subjects (50%) required the administration of vasopressors (phenylephrine hydrochloride) to maintain adequate values of perfusion pressures during surgery, mainly after the induction of anesthesia; the median dose of phenylephrine hydrochloride used was 400 μg/kg/min (IQR: 200, 1,000) (Table 2). The intra- and postoperative median values of serum creatinine, sodium, chloride, and potassium were within normal ranges (reported in Table 2).

**Table 2:** Perioperative variables.

**Perioperative variables**	**Intraoperative** **(*N* = 13)**	**Postoperative** **(*N* = 13)**
**Intraoperative variables**		
Phenylephrine hydrochloride use, *N* (%)	6 (50.0%)	NA
Phenylephrine hydrochloride, μg/Kg/min, median [IQR]	400 [200, 1,000]	NA
Estimated blood loss, mL, median [IQR]	500 [50, 650]	NA
Intraoperative diuresis, mL, median [IQR]	418 [300, 581.3]	NA
Fluid balance intake	3,200 [2,350, 3,950]	NA
Fluid balance output	2,275 [1,815, 2,863]	NA
**Laboratory, unit, median [IQR]**		
Creatinine, mg/dL	0.8 [0.6, 0.9]	0.8 [0.7, 0.9]
Sodium, mmol/L	60 [58.5, 60]	60 [60, 60]
Chloride, mmol/L	138 [136, 140]	135.5 [135, 138]
Potassium, mmol/L	105 [102.5, 107]	103.5 [101, 105]

*N, number; %, percentage; μg, microgram; kg, kilogram; min, minute; mL, milliliter; IQR, interquartile range; NA, not applicable; mg/dL, milligram per deciliter; mmol/L, millimol per liter*.

The overall incidence of major and minor postoperative complications was 33.3% (*n* = 4) and 16.7% (*n* = 2), respectively. Two subjects (16.7%) had a moderate surgical wound infection, two subjects (16.7%) experienced a mild surgical wound infection (did not require surgical drainage and subsided with antibiotic therapy). In addition, one subject (7.6%) developed a major postoperative complication with atrial fibrillation in conjunction with moderate hemorrhaging that required two units of blood transfusion. All the patients were discharged in stable conditions, nine subjects (75%) were discharged home, while three subjects (25%) were discharged to nursing facilities. The readmission rate at 30 days after discharge was 16.7 % and no deaths were reported (Table 1).

## Discussion

This article provides the first-ever retrospective observational cohort study that assessed the incidence of perioperative complications and analyzed clinical outcomes in subjects with established ileostomy scheduled to undergo elective non-ileostomy-related major abdominal surgery under general anesthesia lasting more than 2 h. The incidence of postoperative complications in our cohort was 46.1%. The postoperative complications that we found in our study (surgical wound infection, acute bleeding, and atrial fibrillation) could not be directly attributed to the existence of an established ileostomy at the time of the surgeries of the subjects. Two subjects (16.7%) were readmitted within 30 days of discharge for the management of untreatable pain due to an extension of underlying malignancy. Furthermore, the reported incidence of postoperative complications after extensive abdominal surgeries could reach up to 45%, especially in patients undergoing surgery due to gastrointestinal, hepatobiliary, and/or pancreatic malignancies (17–20).

In a recent retrospective cohort study, Li et al. reported a significant association between ileostomy creation, the onset of AKI, and progression to CKD in patients undergoing colorectal cancer surgery (14). In addition, the authors identified ileostomy as an important predictor for AKI-related readmissions (Odds ratio [OR]: 10.3; 95% CI: 3.9–27.2) and severe CKD after a year (OR: 4.1; 95% CI: 1.4–11.9) (14). Likewise, this significant association between ileostomy, AKI, and progression to CKD has been reported in other studies (21–23). In our patient cohort, the incidence of postoperative complications was 50% having mild or moderate surgical wound infection, acute bleeding, and/or atrial fibrillation were reported.

Messaris et al. reviewed 603 patients undergoing colorectal surgery and diverting loop ileostomy to identify predictive factors for readmission (7). The main preoperative diagnoses were irritable bowel syndrome (50.9%) and rectal cancer (16.1%) (7). The authors reported a total incidence of readmission at 60 days after surgery of 16.9%, with dehydration being the most common cause (43.1%), especially in those patients receiving diuretics as concomitant medication (7). We found a similar readmission rate at 30 days (16.7%) after surgery in our patient setting. However, the main identified cause of readmission in our study was cancer-related severe pain associated with the progression of the malignant disease.

Dehydration, electrolyte alterations, infections, obstruction, prolapse, hernias, AKI, and stoma-related complications are commonly found in patients with established ileostomies (12, 14, 24–27). Colon resections and ileostomy creation may lead to volume depletion, subsequent acid-base and electrolyte instability. Most of these complications usually occur within the first 2 postoperative months (26, 28). Additionally, the time of the ileostomy has been directly associated with an increased risk of enteral ostomy-related complications (25, 29, 30). Even though the median time elapsed from the ileostomy creation and the non-ileostomy surgery in our study was 17.7 months (IQR: 8.3, 32.6), we found no evidence of postoperative ostomy-related complications (e.g., HOS), electrolyte imbalance, and impaired renal function in our patient setting. However, we should consider that due to the median time elapsed from the ileostomy creation and the non-ileostomy targeted surgery on this study of 17.7 months, our sample population had a more robust compensated physiological state regarding intravascular volume depletion and electrolyte imbalance in comparison with patients assessed in other studies during the acute period of ileostomy adaptation (2 months after ileostomy creation). Additionally, 50% of our patients required an intraoperative administration of vasopressors to maintain their hemodynamic stability, mainly during the induction of anesthesia. These episodes of hypotension could be attributed to the combined effect of anesthesia-related effects on cardiovascular function (e.g., heart contractility, arterial/venous vasodilation) in addition to occult hypovolemia, a frequent finding in patients with long-term ileostomies (31, 32). Anesthesia-induced venodilation is widely recognized as one of the main causes of relative hypovolemia, increasing venous compliance ensuing a subsequent decrease in venous return, preload, and response to vasopressors (33). This temporary state of relative hypovolemia could be clinically undetected by anesthesia care providers, resulting in potential oxygen delivery and/or tissue perfusion impairment (33). Therefore, it is important to recognize the presence of occult intravascular volume depletion and correct it, as a measure to attenuate the cardiovascular impact of the anesthetic drugs and reduce the incidence of intraoperative hypotension. In a recent publication, Ejaz et al. (34) identified intra- and post-operative major blood loss requiring blood transfusion, and tachypnea as perioperative risk factors linked to postoperative surgical site infection (SSI) after major abdominal surgery, while other common clinical situations like intraoperative hypothermia, hyperthermia, bradycardia, tachycardia, hypotension, and hypertension were not associated with SSIs. Babazade et al. (35) retrospectively studied 2,531 patients who underwent colorectal surgery and did not find any correlation between intraoperative hypotension and SSI after colorectal surgery. Yilmaz et al. (36) conducted a retrospective, cohort study in 5,896 patients who sustained colorectal surgery and concluded that postoperative hypotension was not associated with SSI. Conversely, the two subjects (16.7%) that developed moderate surgical wound infection did not present with sustained intraoperative episodes of hypotension or recorded hypotension during PACU or ward hospitalization, while the patient that experienced atrial fibrillation after postoperative bleeding that required blood transfusion did not develop a surgical wound infection.

A recent meta-analysis conducted by Gavriilidis et al. compared the incidence of SSI among patients that underwent loop transverse colostomy (*N* = 628) vs. loop ileostomy (*N* = 906) (37). The study found a lower incidence of SSI among the subjects that underwent loop ileostomy (1%; 8/575; *p* < 0.001) when compared with the loop transverse colostomy group (5%; 14/299) (37). On the other hand, the shaving or constant pulling of the hair adhesives around the stoma during the replacement of the appliances leads to the presence of folliculitis (caused by Staphylococcus aureus) that could trigger the postoperative SSI complication in this vulnerable surgical population (38, 39). Our study showed a high incidence (33.4%) of SSI (mild or moderate) that might be related to the aforementioned peristomal skin complication.

The most common comorbidity found in our study was psychiatric disorders (58.3%). This finding is supported by robust evidence that showed an increased incidence of psychosocial conditions in individuals with a stoma, which has a negative impact on their quality of life (40–42).

A retrospective study conducted by Vergara-Fernandez et al. assessed the potential predictors of high output ileostomy-related complications (1). The study included 102 adult patients undergoing colorectal surgery with primary low pelvic anastomosis and diverting loop ileostomy at elective or emergency settings. The authors concluded that patients with a history of ulcerative colitis and those with a current ileostomy output >1 L/day at discharge were more likely to develop high output-related complications (1).

We acknowledge the limitations in our study that should be considered. First, the single-center and retrospective design of the study limited our analysis, as well our ability to identify reliable statistical significance in the primary and secondary objectives. Second, the small sample size (12 patients) may increase the probability of a Type II error biasing the accuracy of our findings. Third, and perhaps the most significant limitation, we found a high variability of the median time from the creation of ileostomy to surgery date (17.7 months, IQR: 8.3, 32.6). This could have had an important impact on our findings considering that most of the ileostomy-related complications occur during the first 12 months after ileostomy creation. Lastly, certain limitations linked to the retrospective nature of our study (e.g., data not collected and/or measured) should also be considered.

## Conclusions

The perioperative management of patients with established ileostomies undergoing major abdominal surgeries might be challenging to anesthesia care providers and surgeons. Despite AKI and dehydration is the most common complications after ileostomy is formed, occult hypovolemia and electrolyte imbalance assessment must be treated and/or corrected prior to anesthesia induction to avoid other short- and long-term complications, regardless of the active length of the functioning ileostomy. Hence, hemodynamic and laboratory parameters should be closely monitored throughout the perioperative period. Our findings suggest that the presence of a well-established (longer than a year) ileostomy might not represent a relevant risk factor for significant perioperative complications related to fluid management or hospital readmission in patients undergoing abdominal surgery unrelated to a functioning ileostomy. However, the presence of peristomal skin complications could trigger a higher incidence of surgical wound infection. Nevertheless, we are not able to form robust conclusions due to the small sample size; thus, future prospective trials focusing on short-term outcomes and complications in this patient setting may better identify the pros and cons of different perioperative approaches.

## Data Availability Statement

The raw data supporting the conclusions of this article will be made available by the authors, without undue reservation.

## Ethics Statement

The studies involving human participants were reviewed and approved by Office of Responsible Research Practices—The Ohio State University. Written informed consent for participation was not required for this study in accordance with the national legislation and the institutional requirements.

## Author Contributions

AU, TW, ME-V, JF-D, and LL: conceptualization. AU, ME-V, LP, HS, JF-D, and AG-Z: data curation. AU, ME-V, and MA-R: formal analysis. AU, TW, ME-V, LP, HS, JF-D, AG-Z, and LL: investigation. AU, LP, HS, and LL: methodology. AU, TW, and LL: project administration, resources, supervision, and visualization. AU, TW, MA-R, and LL: validation. AU, TW, ME-V, and JF-D: writing—original draft. AU, TW, ME-V, LP, HS, JF-D, AG-Z, MA-R, and LL: writing review and editing. All authors contributed to the article and approved the submitted version.

## Conflict of Interest

The authors declare that the research was conducted in the absence of any commercial or financial relationships that could be construed as a potential conflict of interest.

## Publisher's Note

All claims expressed in this article are solely those of the authors and do not necessarily represent those of their affiliated organizations, or those of the publisher, the editors and the reviewers. Any product that may be evaluated in this article, or claim that may be made by its manufacturer, is not guaranteed or endorsed by the publisher.

## Abbreviations

HOS: high-output stoma
IBD: inflammatory bowel disease
AKI: acute kidney injury
CKD: chronic kidney disease
ASA: American Society of Anesthesiologists
BMI: body mass index
EBL: estimated blood loss
PACU: post-anesthesia care unit
ICU: intensive care unit
IQR: Interquartile Range
CONSORT: Consolidated Standards of Reporting Trials
LOS: length of hospital stay
OR: Odds ratio
SSI: surgical site infection.
